# Genome-Wide Screening and Stability Verification of the Robust Internal Control Genes for RT-qPCR in Filamentous Fungi

**DOI:** 10.3390/jof8090952

**Published:** 2022-09-10

**Authors:** Yayong Yang, Xinyu Xu, Zhuohan Jing, Jun Ye, Hui Li, Xiaoyu Li, Lei Shi, Mengyu Chen, Tengyun Wang, Baogui Xie, Yongxin Tao

**Affiliations:** 1 College of Horticulture, Fujian Agriculture and Forestry University, Fuzhou 350002, China; 2 Mycological Research Center, College of Life Sciences, Fujian Agriculture and Forestry University, Fuzhou 350002, China; 3 Institute of Cash Crops, Hebei Academy of Agriculture and Forestry Sciences, Shijiazhuang 050051, China

**Keywords:** internal control genes, filamentous fungi, RT-qPCR, gene expression

## Abstract

In real-time quantitative PCR (RT-qPCR), internal control genes (ICGs) are crucial for normalization. This study screened 6 novel ICGs: Pre-mRNA-splicing factor cwc15 (*Cwf15*); ER associated DnaJ chaperone (*DnaJ*); E3 ubiquitin-protein ligase NEDD4 (*HUL4*); ATP-binding cassette, subfamily B (MDR/TAP), member 1 (*VAMP*); Exosome complex exonuclease DIS3/RRP44 (*RNB*); V-type H^+^-transporting ATPase sub-unit A (*V-ATP*) from the 22-transcriptome data of 8 filamentous fungi. The six novel ICGs are all involved in the basic biological process of cells and share the different transcription levels from high to low. In order to further verify the stability of ICGs candidates, the six novel ICGs as well as three traditional housekeeping genes: *β*-actin (*ACTB*); *β*-tubulin (*β-TUB*); glyceraldehyde-3-phosphate dehydrogenase gene (*GAPDH*) and the previously screened reference genes: SPRY-domain-containing protein (*SPRYp*); Ras-2 protein (*Ras*); Vacuolar protein sorting protein 26 (*Vps26*) were evaluated by geNorm and NormFinder statistical algorithms. RT-qPCR of 12 ICGs were performed at different developmental stages in *Flammulina filiformis* and under different treatment conditions in *Neurospora crassa*. The consistent results of the two algorithms suggested that the novel genes, *RNB*, *V-ATP*, and *VAMP*, showed the highest stability in *F. filiformis* and *N. crassa*. *RNB*, *V-ATP*, and *VAMP* have high expression stability and universal applicability and therefore have great potential as ICGs for standardized calculation in filamentous fungi. The results also provide a novel guidance for the screening stable reference genes in RT-qPCR and a wide application in gene expression analysis of filamentous fungi.

## 1. Introduction

Real-time quantitative PCR (RT-qPCR) has been widely used in molecular biology research [1] due to its accurate, sensitive, rapid, and economical characteristics [2,3], which has become an effective method for quantitative transcription level analysis of target gene [4,5]. In RT-qPCR, the amount of PCR product formed is proportional to the fluorescence of dyes introduced into the reaction, and the number of amplification cycles required to obtain a particular amount of DNA molecules is registered [6]. The accuracy of RT-qPCR results depends on many factors, including the quality and quantity of initial RNA, primer specificity, amplification efficiency, etc. [7]. Thus, normalization of RT-qPCR using appropriate internal reference genes as internal controls is important to eliminate the impact of differences in RNA yield (due to variation in extraction), cDNA yield (due to variation in reverse-transcription), and amplification efficiency on gene expression levels [8]. Ideally, internal reference genes must display constitutive, stable expression in all cell types/tissues and different experimental treatments [9]. In reality, no gene in the cell can satisfy the condition of completely constant expression in any time and space conditions. The previous studies often used the well-known housekeeping genes as internal reference genes, such as *β*-actin (*ACTB*); *β*-tubulin (*β-TUB*); glyceraldehyde-3-phosphate dehydrogenase gene (*GAPDH*) and 18S rRNA (*18S*) [10,11,12,13]. However, evidence has increasingly shown that these traditional reference genes cannot be expressed stably and they even have large differential expressions with the popularity of high-throughput sequencing in recent years. For example, the expression level of *GAPDH* was reported to vary depending on the organism, tissue, diseases, and many other factors in many species, such as *Ganoderma lucidum*, *Volvariella volvacea,* and *Flammulina filiformis* [14,15,16]. *ACTB* is involved in cell movement, cell structure, and cell integrity, so it may be expressed differently under conditions, such as starvation or growth factor stimulation [15,17]. A drawback to the use of 18S rRNA as a reference gene is imbalance between mRNA and rRNA fractions. As with *GAPDH* and *ACTB*, 18S rRNA has been shown to be regulated in response to various biological stimuli [9], and it is not suitable as an internal reference gene for normalization. Therefore, it is of significance to screen novel stable and universal internal control genes (ICGs).

In previous studies, there were also some studies that screened several new internal reference genes. In *G. lucidum*, cyclophilin (*CYP*), and translation initiation factor (*TIF*) were found to be the most stable genes under heat stress condition [18]. In *Cordyceps militaris*, polymerase II large subunit (*rpb1*) was showed to be the best reference gene during the developmental stages [19]. In *V. volvacea*, three novel ICGs: SPRY-domain-containing protein (*SPRYp*); Ras-2 protein (*Ras*); Vacuolar protein sorting protein 26 (*Vps26*) were evaluated and exhibited the highest expression stability [15]. However, these newly screened reference genes are not always applicable in new species or under new conditions. For example, *SPRYp* and MSF1-domain-containing protein (*MSF*) which were screened from *V. volvacea* and stably expressed in developmental stages showed the least stable expression under different stresses, substrates, and developmental stages in *Lentinula edodes* [20]. This may be due to previous studies on internal reference genes being limited to single species or being limited under several specific experimental conditions. Such as in *Morchella importuna*, peptidyl-prolyl cis-trans isomerase (*CYC3*) was the most stable gene in different development stages and vacuolar ATP synthase (*INTF4*) and Elongation factor 1-alpha (*AEF3*) were the top-ranked genes across carbon sources, while vacuolar protein sorting (*INTF3*) and *CYC3* showed the robust stability for temperature stress treatment [21]. Every special screening ICG for a particular species and experimental condition results in additional experimental content and tedious validation when a new RT-qPCR experiment is carried out. Therefore, it is very valuable and practical to screen some more general and universal stable ICGs.

In this study, genome-wide transcriptome data from several filamentous fungi including three basidiomycetes and five ascomycetes were used to screen the universal stable ICGs. The absolute RPKM values and function of the novel screened ICGs were also analyzed. At the same time, *F. filiformis* and *N. crassa* were selected as the representatives of basidiomycetes and ascomycetes, respectively, to verify their expression stability. *F. filiformis* with the typical morphological and developmental characteristics of fruiting body of agaric fungi, has completed genome sequencing and transcriptome sequencing for its different developmental stages. While *N. crassa* is easy to operate in genetics and has been studied in depth as a model ascomycete. In total, 10 samples in different developmental stages of *F. filiformis* and the 8 samples under different conditions of *N. crassa* make sure that the samples for stability validation have high diversity and representativeness. The expression stability of these ICGs candidates was further evaluated using the professional geNorm and NormFinder statistical algorithms. The results will provide some novel, reliable and universal stable ICGs for RT-qPCR research in filamentous fungi.

## 2. Materials and Methods

### 2.1. Strains and Culture Conditions

*F. filiformis* dikaryotic strain FL19 was obtained from the Fujian Edible Fungi Germplasm Resource Collection Center of China and maintained with periodic transfers on potato dextrose agar (PDA) at 25 °C. *N. crassa* wild-type strain FGSC#4200 (WT) and the *NcSpds* gene deletion mutant strain FGSC#18934 (∆Spds) were provided by the Institute of Microbiology, Chinese Academy of Sciences and maintained with periodic transfers on Vogel’s solid medium [22] at 25 °C.

The 10 samples from different developmental stages of *F. filiformis* and 8 samples of *N. crassa* under different environmental factors were used for the stability verification of ICGs candidates. *F. filiformis* FL19 mycelia (MY) were collected after cultivating on PDA plates for 7 d at 25 °C in the dark. The fruiting body of the FL19 strain was cultivated via the method described by Tao et al. [23]. Aerial hyphal knot (AHK) was collected within 2–5 d before primordium formation, when the mycelia gathered into small knots. Fruiting body of the FL19 strain was sampled at five different developmental stages: primordia (PR), bud (BUD), pileus in young fruiting body (YFP), stipe in the young fruiting body (YFS), pileus in elongation stage (ELP), stipe in elongation stage (ELS), pileus in maturation stage (MAP), and stipe in maturation stage (MAS). *N. crassa* WT and ∆Spds strains were cultured on Vogel’s solid medium coated with cellophane for four days at 25 °C in the dark. The WT strain overgrown with plates was treated under cold stress (CS) at 4 °C for 30 min, heat stress (HS) at 42 °C for 30 min, and blue light (3 μmoL·m^−2^·s^−1^) stress (BS) for 3 h, respectively. In addition, *N. crassa* WT strain was also subjected to continuous blue light (3 μmoL·m^−2^·s^−1^) treatment (BT) and was also cultured in Vogel’s solid medium with 0.01 mmol/L JA and 5 mmol/L H_2_O_2_, respectively. All samples were snap-frozen in liquid nitrogen after collection and stored at −80 °C.

### 2.2. Screening for Novel ICGs

A total of 22 sets of gene expression profiles data from eight fungi (three basidiomycetes: *F. filiformis*, *Agaricus bisporus* and *V. volvacea*, five ascomycetes: *N. crassa*, *Trichoderma reesei*, *Fusarium graminearum*, *Magnaporthe oryzae* and *Aspergillus nidulans*) were obtained from Gene Expression Omnibus (GEO) database (https://www.ncbi.nlm.nih.gov/, accessed on 1 November 2021). They were divided into four groups according to sample types: the different nutritional resources, the different development stages, the different stresses and the different strains. The OrthoFinder and double-Blastp were used to determine the relationship of orthologous genes among the eight filamentous fungi, the genome data of which were downloaded from the Joint Genome Institute (JGI) database (https://jgi.doe.gov/, accessed on 1 November 2021). According to the orthologous genes corresponding to eight filamentous fungi, the RPKM values of each gene were obtained from the RNA-Seq data in each experiment by the VLOOKUP formula. The average RPKM value, maximum and minimum of RPKM value and the maximum fold change (MFC) were calculated by excel formulas in each experiment and further analyzed for the expression stability of each gene.

### 2.3. Function Analysis of ICGs Candidates

The function of 12 ICGs candidates was analyzed in this study. Three traditional ICGs (*ACTB*, *β-TUB* and *GAPDH*) commonly used as ICGs in *Aspergillus flavus* [24], *Candida tropicalis* [25], *Lentinula edodes* [26] and three ICGs candidates *(SPRYp*, *Ras*, *and Vps26)* screened from *V. volvacea* by Tao et al. [15] were identified in the genome of *F. filiformis* L11 (GenBank Accession No. APIA00000000.1; BioProject: PRJNA191865) and *N. crassa* OR74A (GenBank Accession No. GCA_000182925.2; BioProject: PRJNA13841) confirmed by cross BLAST searches in NCBI. Novel six ICGs candidates: Pre-mRNA-splicing factor cwc15 (*Cwf15*); ER associated DnaJ chaperone (*DnaJ*); E3 ubiquitin-protein ligase NEDD4 (*HUL4*); ATP-binding cassette, subfamily B (MDR/TAP), member 1 (*VAMP*); Exosome complex exonuclease DIS3/RRP44 (*RNB*); V-type H^+^-transporting ATPase sub-unit A (*V-ATP*) were screened from 22 sets of RNA-Seq data in 8 common filamentous fungi based on their expression levels and function. The corresponding accession number of 12 ICGs in *F. filiformis* and *N. crassa* was listed in Table S2 (Supplementary Material).

The amino acid sequences of the 12 ICGs candidates were extracted from the *F. filiformis* protein database. The amino acid sequences of the 12 ICGs candidates were uploaded to the Pfam database (https://pfam.xfam.org/, accessed on 3 March 2022) to predict their functions, respectively.

### 2.4. Total RNA Extraction and RT-qPCR

Total RNA was extracted using FastPure^®^ Plant Total RNA Isolation Kit (Vazyme Biotech, Nanjing, China). The extracted RNA samples were quantified with a NanoND-1000 spectrophotometer (NanoDrop Technologies, Wilmington, DE, USA), and the RNA concentration and ratios of A260 and A280 were obtained simultaneously. Only RNA samples with A260/A280 values between 1.9 and 2.1 were used for cDNA synthesis. The total RNA concentration extracted from each sample was diluted to 500 ng/µL with RNase-free ddH_2_O. The 2 μL (1000 ng) RNA diluent was used to synthesize cDNA for each sample, according to the instructions of the HiScript^®^ III All-in-one RT SuperMix Perfect for qPCR (Vazyme Biotech, Nanjing, China), with gDNA wiper.

The primers for RT-qPCR were designed in the coding sequence (CDS) region of target genes using the PrimerQuest^™^ Tool (https://sg.idtdna.com/PrimerQuest/Home/Index, accessed on 5 March 2022) online website, and the primer design refer to the principles of Alicia Rodríguez et al. [27]. The length of primer and the length of amplification product were set as 20–30 bp and 100–250 bp, respectively. The melting temperature (T_m_) of primers were set to 45–65 °C. All primers were detected by OligoAnalyzer^™^ Tool online website (https://sg.idtdna.com/calc/analyzer, accessed on 5 March 2022) for GC content, melting temperature (T_m_), self-complementary, primer-dimer and hairpin formation, degree of degeneracy, 5′ end stability, and 3′ end specificity. All primers were listed in Tables S3 and S4 (Supplementary Material).

RT-qPCR was performed using a CFX96 Real-Time PCR Detection System (Bio–Rad, Hercules, CA, USA). RT-qPCR amplification included a denaturation step of 30 s at 95 °C, followed by 40 cycles of 10 s at 95 °C and 30 s at primer-specific annealing temperatures, according to the instructions of Taq Pro Universal SYBR qPCR Master Mix (Vazyme Biotech, Nanjing, China). In addition, the amplification efficiency of all RT-qPCR primers was required between 90% and 110%. The R^2^ of the standard curve was all greater than 0.98, and the standard deviation of the Ct values of the three technical replicates of each sample were all less than 0.2. The above processes were performed strictly to ensure that RT-qPCR met the MIQE standard, recommended by Bustin et al. [28,29].

### 2.5. Data Processing and Statistical Analysis

The RT-qPCR cycle threshold (Ct) value of each ICG candidate was obtained by calculating the arithmetic mean from three technological and three biological replicates. The arithmetic mean Ct value were processed and plotted by the prism program in GraphPad 9.0 software, and converted to Q value with the 2^−∆Ct^ method for geNorm and NormFinder analyses. The geNorm version 3.5 [30] and NormFinder version 0.953 [31], were used to evaluate the expression stabilities of the ICGs candidates.

## 3. Results

### 3.1. Several Traditional Housekeeping Genes Frequently Show Instability

In the previous experiments for the relative expression of genes, several traditional housekeeping genes which were often used for normalized calculation in RT-qPCR includes 18S rRNA (*18S*), *β*-actin (*ACTB*), cyclophilin (*CYP*), glyceraldehyde-3-phosphate dehydrogenase (*GAPDH*), histone H3 (*HH3*), *β*-tubulin (*β-TUB*), ubiquitin (*UBQ*), etc. Here, we randomly selected the three most common and popular genes (*ACTB*, *β-TUB*, and *GAPDH*) to test their stability and applicability in multiple transcriptome data. There are 22 sets of gene expression profiles obtained from RNA-Seq from 8 common filamentous fungi (Table 1). In these profiles, the expression level of all genes in each sample was showed as the RPKM value, and the ratio of the maximum and minimum RPKM (the maximum fold change, MFC) among samples in each experimental set was used to evaluate their expression stability. As shown in Table 1, the MFC value of *ACTB* gene was between 1.1 and 3.7 and was more than 2 and 3 in 8 sets (36.4%) and 1 set (4.5%), respectively. The MFC value of *β-TUB* gene was between 1.4 and 5.3, and was more than 2 and 3 in 11 sets (50.0%) and 4 sets (18.2%), respectively. The MFC value of *GAPDH* gene was between 1.4 and 19.7, and was more than 2 and 3 in 16 sets (72.7%) and 11 sets (50.0%), respectively. The three traditional housekeeping genes all showed varying degrees of instability under the criteria of MFC ≤ 2 (strict) or ≤ 3 (moderate), which means that they are not suitable as ICGs for normalized calculation in RT-qPCR.

We can also find that the instability of housekeeping genes occurred in the most cases of different nutritional resources and development stages from Table 1. As far as the absolute value of transcription level of the three traditional housekeeping genes, the RPKM of *ACTB* gene was in the range of 500 to 3550 (the average value was 1479.5); the RPKM of *β-TUB* gene was in the range of 200 to 4500 (the average value was 1274.2); the RPKM of *GAPDH* gene was in the range of 800 to 50,000 (the average value was 11,115.7). The average RPKM values of the housekeeping genes are relatively high, which is not suitable for normalization of low expression genes.

### 3.2. Stable ICGs Identified in the Literature Are Not Universally Applicable

Previous studies focused more on comparing the stability of different traditional housekeeping genes, and less on screening for novel stable reference genes. In *V. volvacea*, we previously identified three novel stable reference genes: *SPRYp*, *Ras*, and *Vps26* (Tao et al., 2016). Here, the three reference genes were also evaluated in 22 sets of gene expression profiles data. As shown in Table 1, the RPKM of *SPRYp* gene was in the range of 18 to 150 (the average value was 66.9); the RPKM of *Ras* gene was in the range of 8 to 500 (the average value was 126.8); the RPKM of *Vps26* gene was in the range of 16 to 100 (the average RPKM was 48.0). Their relatively low RPKM values make them potentially useful as internal reference for RT-qPCR of low abundance expressed genes. The MFC value of *SPRYp* gene was between 1.0 and 10.9, and was more than 2 and 3 in 12 sets (54.5%) and 8 sets (36.4%), respectively. The MFC value of *Ras* gene was between 1.1 and 4.3 and was more than 2 and 3 in 9 sets (40.9%) and 6 sets (27.3%), respectively. The MFC value of *Vps26* gene was between 1.1 and 3.0 and was more than 2 and 3 in 4 sets (18.2%) and 0 sets (0), respectively. As with traditional housekeeping genes, we can also find from Table 1 that the three novel reference genes: *SPRYp*, *Ras*, and *Vps26* also showed the instability mainly under different nutritional resources or developmental stages with varying degrees of instability under the criteria of MFC ≤ 2 (strict) or ≤ 3 (moderate, except *Vps26*). It means that they do not have broad universal applicability as ICGs in RT-qPCR.

### 3.3. Screening of Novel Stable ICGs in Multiple Species

To screen novel universal and stable ICGs, we screened the candidate stably expressed genes by using the criteria of MFC ≤ 3 (moderate) from the above 22 sets of gene expression profiles data in 8 common filamentous fungi. In total, 533 genes were showed stable expression firstly, then 50 genes remained after excluding predicted genes, hypothetical genes, genes of unknown function, and highly similar gene families. Finally, we selected six novel candidates based on the average RPKM value at different levels: low level (the average RPKM < 100: *Cwf15*, *DnaJ*), medium level (100 < the average RPKM < 300: *HUL4*, *VAMP*), and high level (the average RPKM > 300: *RNB*, *V-ATP*) (Table 2). As shown in Table 2, the MFC value of *DnaJ* gene was between 1.1 and 2.0, and was less than 2 in all sets. The MFC value of *Cwf15* gene was between 1.2 and 3.0 and was more than 2 in four sets (18.2%). The MFC value of *VAMP* gene was between 1.1 and 2.4, and was more than 2 in four sets (18.2%). The MFC value of *HUL4* gene was between 1.1 and 2.3, and was more than 2 in two sets (9.1%). The MFC value of *RNB* gene was between 1.1 and 3.0, and was more than 2 in four sets (18.2%). Moreover, the MFC value of *V-ATP* gene was between 1.1 and 2.8, and was more than 2 in four sets (18.2%). The MFC value of the six novel candidates was less than 3 (MFC ≤ 3) in all sets and less than 2 with the percentage of 81% or more. The results suggested that these six genes have high expression stability and universal applicability in filamentous fungi, so they have great potential as internal reference for normalized calculation in RT-qPCR.

### 3.4. Identification and Functional Analysis of Novel Screened Stable ICGs

The protein structure and function of the six novel ICGs candidates as well as three traditional housekeeping genes and three stable reference genes screened in *V. volvacea*, were analyzed through Pfam database (Table 3).

Cwf15 is a component of the Cef1 complex, and forms part of the spliceosome, which is thought to be involved in pre-mRNA splicing. VAMP has endoplasmic reticulum (ER) proteins (VAPs) domain, which plays crucial roles in vesicle trafficking, neurotransmitter release, microtubule organization, lipid transport, and unfolded protein response. DnaJ is able to stimulate the ATPase activity of hsp70 and has been shown to bind both L-peptides and d-peptides, and it may be interacted with the side chain of the substrate. RNB has domain of the DIS3-like exonuclease 2 (DIS3L2) that specifically recognizes RNAs polyuridylated at their 3’ end and also plays an important role in the mRNA degradation pathway. HUL4 has three domains: modular protein domain (WW domain), homologous to the E6-AP Carboxyl Terminus (HECT domain) and Ca^2+^-dependent membrane-targeting module (C2 domain). The WW domain can mediate specific interactions with protein ligands; C2 domain is involved in signal transduction or membrane transport; and HECT domain is involved in E2 binding. V-ATP drives proton pumps and is involved in a variety of important intracellular and intercellular processes, such as receptor-mediated endocytosis, protein trafficking, active transport of metabolites, homeostasis, and neurotransmitter release.

Similar to traditional housekeeping genes and the previously screened reference genes, these six novel candidates are generally involved in the basic biological processes of cells and share the high conserved function among the different species, which make them have the potential as the stable and universal ICGs.

### 3.5. Mean and Dispersion of Ct values of Novel Screened ICGs in F. filiformis and N. crassa

**Figure 1:**
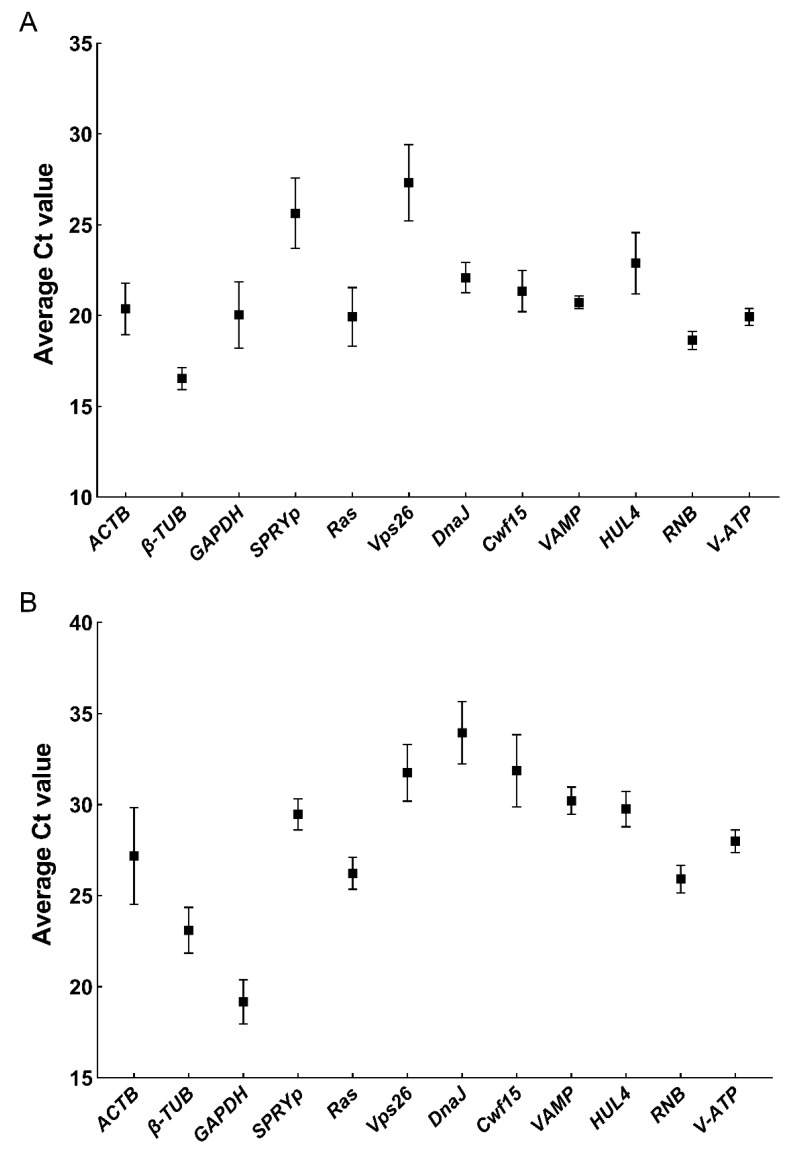
Variation in RT-qPCR value of 12 ICGs in samples of *F. filiformis* and *N. crassa*. (**A**) Average Ct value of each ICGs in 10 samples of *F. filiformis* (10 developmental stages samples of in the *F. filiformis* dikaryotic strain FL19: mycelia, aerial hyphal knot, primordia, bud, pileus in the young fruiting body, stipe the in the young fruiting body, pileus in elongation stage, stipe in elongation stage, pileus in maturation stage and stipe in maturation stage) were shown on the y-axis. The 12 ICGs candidates were shown on the x-axis. Error bars represent the maximum Ct value and the minimum Ct value, respectively. (**B**) Average Ct value of each ICGs in eight samples of *N. crassa* (WT; ∆Spds; BT; BS; CS; HS; JA; H_2_O_2_) were shown on the y-axis. The 12 ICGs candidates were shown on the x-axis. Error bars represent the maximum Ct value and the minimum Ct value, respectively.

**Figure 2:**
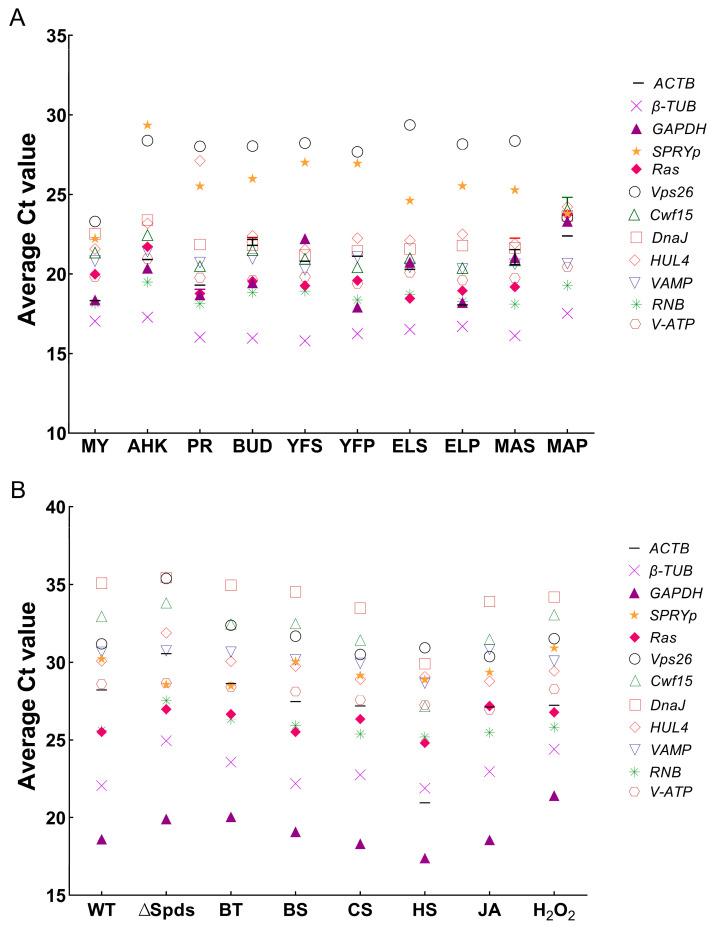
RT-qPCR Ct value of 12 ICGs in different samples of *F. filiformis* and *N. crassa*. (**A**) Average Ct value of three biological replicates and three technical replicates were shown on the Y-axis. The 10 samples of different developmental stages in *F. filiformis* were shown on the X-axis: mycelia, aerial hyphal knot; primordia, bud, pileus in the young fruiting body, stipe in the young fruiting body, pileus in elongation stage, stipe in elongation stage, pileus in maturation stage and stipe in maturation stage. (**B**) Average Ct value of three biological replicates and three technical replicates were shown on the Y-axis. The eight samples of different treatment conditions in *N. crassa* were shown on the X-axis: WT, ∆Spds, BT, BS, CS, HS, JA, and H_2_O_2_.

To further confirm the stability and superiority of the novel screened ICGs candidates, the discreteness of Ct values of RT-qPCR was performed in two typical filamentous fungi: *F. filiformis* (model basidiomycete) and *N. crassa* (model ascomycete). Overall, the stability of 12 ICGs candidates were irregularly distributed in basidiomycetes and ascomycetes expression data (Figure 1 and Figure 2). In Figure 1A, Ct value of *VAMP* gene showed the smallest fluctuation range (1.51) in *F. filiformis*, followed by *RNB* (1.56) and *V-ATP* (2.00). The Ct value of *SPRYp* gene showed the largest fluctuation range (7.31) in *F. filiformis*, followed by *Vps26* (6.26) and *HUL4* (5.99). Usually, the smaller fluctuation range of Ct value means the more stable expression. Thus, the top three stable ICGs were *VAMP*, *RNB* and *V-ATP* in *F. filiformis*, according to the simple rough comparison of Ct value fluctuation. In Figure 1B, Ct value of *V-ATP* gene showed the smallest fluctuation range (1.84) in *N. crassa*, followed by *VAMP* (2.69) and *RNB* (2.70). The Ct value of *ACTB* gene showed the largest fluctuation range (9.99) in *N. crassa*, followed by *Cwf15* (7.18) and *DnaJ* (6.25). Thus, the top three stable ICGs were *V-ATP*, *VAMP* and *RNB* in *N. crassa*, according to the results of Ct value fluctuation. Taken together, *VAMP*, *V-ATP* and *RNB* gene had small fluctuation range in both *F. filiformis* and *N. crassa*.

In addition, the average Ct value can also reflect the absolute expression level of the gene, based on the principle that the expression level for one gene is inversely proportional to its Ct value. In Figure 2A, *Vps26* has the largest average Ct value (27.31), in 10 developmental stages of *F. filiformis*, meaning its expression level is the lowest among these 12 ICGs candidates. While *β-TUB* showed the smallest average Ct value (16.52) and the highest expression level in *F. filiformis* life cycle. In Figure 2B, *DnaJ* has the largest average Ct value (33.94) in 8 samples of different treatment conditions of *N. crassa*, meaning its expression level is the lowest among these 12 ICGs candidates, while *GAPDH* showed the smallest average Ct value (19.17) and the highest expression level in different conditions of *N. crassa*.

### 3.6. Stability Evaluation of Novel Screened Stable ICGs in F. filiformis and N. crassa

**Figure 3:**
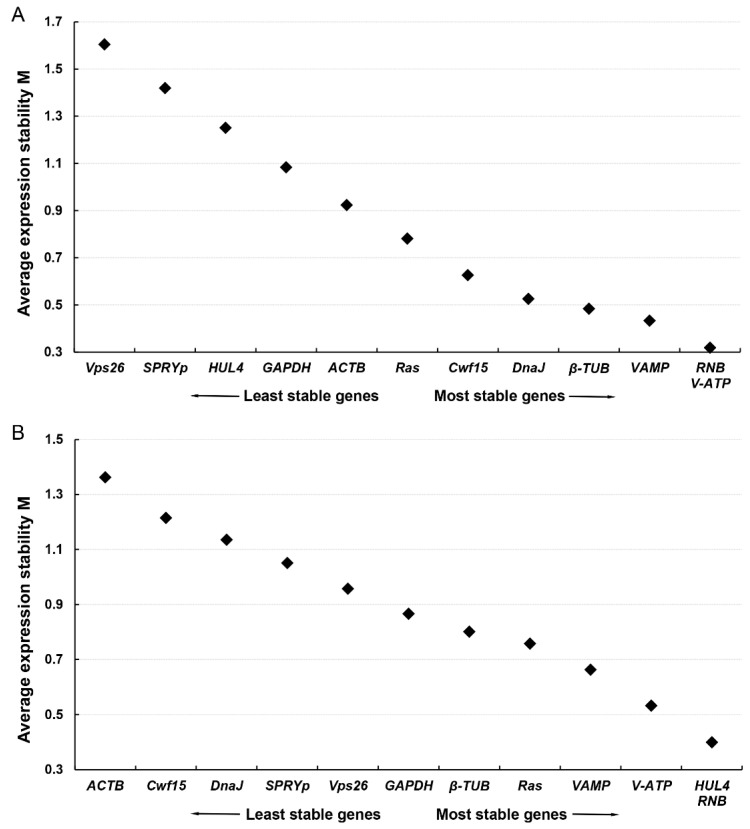
The expression stability ranking of 12 ICGs candidates using geNorm in *F. filiformis* and *N. crassa*. The stability measure M values, calculated by geNorm, were shown on the y-axis. The lowest M values represent the most stable expression. (**A**) The expression stability ranking of 12 ICGs candidates in *F. filiformis*. (**B**) The expression stability ranking of 12 ICGs candidates in *N. crassa*.

The stability of the 12 ICGs candidates was evaluated based on RT-qPCR Ct values using 2 professional statistical algorithms: geNorm and NormFinder. Firstly, the gene expression stability measure M value of each ICG was calculated based on the average pairwise variation by geNorm. The geNorm program arranged the ICGs candidates in descending order according to the M value, and the lower M values (i.e., less average variation) indicate higher expression stability. In *F. filiformis* (Figure 3A), *RNB*, *V-ATP* and *VAMP* were indicated as the most stable genes with the M values of 0.32 and 0.43, respectively, followed by *β-TUB* (M value: 0.48). While, *Vps26*, *SPRYp*, and *HUL4* were indicated as least stable relatively, with the M value range of 1.25–1.65. The geNorm results was consistent with the discreteness of Ct values in Figure 1A. In *N. crassa* (Figure 3B), *RNB*, *HUL4* and *V-ATP* were indicated as the most stable genes with the M values of 0.40 and 0.53, respectively, followed by *VAMP* (M value: 0.66). While, *ACTB*, *Cwf15*, and *DnaJ* were indicated as least stable relatively, with the M value range of 1.05-1.40. The geNorm results was generally consistent with the discreteness of Ct values (Figure 1B) in *N. crassa*. Taken together, *RNB* was thus the most stably expressed gene in all test samples in both *F. filiformis* and *N. crassa* according to geNorm, followed by *V-ATP* and *VAMP*.

NormFinder is another professional statistical algorithm, which uses a model-based approach to rank ICGs based on inter and intra-group expression variations. It calculates the stability value of each gene, and ranks the candidates in descending order based on their stability value. In *F. filiformis* (Table 4), the most stable gene was *RNB* with the stability value of 0.110, followed by *V-ATP* and *VAMP*, with the stability values of 0.114 and 0.353, respectively. The three least stable genes were *Vps26*, *SPRYp* and *HUL4*, with the stability values of 1.610, 1.251 and 1.185, respectively. In *N. crassa* (Table 4), the most stable gene was *RNB* with the stability value of 0.387, followed by *VAMP* and *V-ATP*, with the stability values of 0.398 and 0.454, respectively. The three least stable genes were *ACTB*, *SPRYp*, and *Vps26*, with the stability values of 1.365, 0.948, and 0.835, respectively. The comprehensive results of *F. filiformis* and *N. crassa* showed that *V-ATP* was the most stable gene, with the stability value was 0.175, followed by *RNB*, *β-TUB*, and *VAMP*, with the stability value of 0.261, 0.266, and 0.348, respectively. The four least stable genes were *Vps26*, *ACTB*, *SPRYp*, and *Cwf15*, with the stability value of 1.507, 1.501, 1.426, and 1.350, respectively.

Combining the results of geNorm and NormFinder, three novel genes: *RNB*, *V-ATP*, and *VAMP* showed the highest expression stability in both *F. filiformis* and *N. crassa*, followed by the traditional *β-TUB* gene.

## 4. Discussion

RT-qPCR is an almost inevitable technology to detect the relative expression of genes. What and how many reference genes used for normalization calculation in RT-qPCR is a crucial determinant of the accuracy of expression quantification [34]. According to the needs of practical research, the good ICG should generally meet the following criteria: (1) It should have high expression stability under various conditions. (2) It should have universal applicability in different species, or different strains in one species. (3) Its expression level should be close to that of the target gene (estimated). (4) The function of ICG is conserved among different species, which is beneficial to find its orthologous gene in a new species. In this study, we screened more universally applicable stable ICGs from whole gene expression profile data of common model filamentous fungi. The transcriptomes contain four sample types, and the internal reference genes screened on this basis are more suitable for the analysis of gene expression under different experimental conditions. No gene can meet the requirements of absolutely strict criteria for no difference in expression in any experimental conditions [14], therefore we used MFC < 3 as a mild criterion for stable expression and screened for six different expression levels ICGs candidates. At the same time, 18 samples were verified, including 10 samples of different developmental stages in *F. filiformis* and 8 samples of different stress treatment conditions in *N. crassa*. Different and abundant developmental stages and treatments under different environmental factors increase the diversity and representativeness of samples, which is more conducive to screening stable ICGs. After further analysis of ICGs candidates by the geNorm and NormFinder program, *RNB*, *V-ATP*, and *VAMP* were finally proved to be the most stable expressed ICGs. Their stability was higher than that of the three traditional ICGs (*ACTB*, *β-TUB*, and *GAPDH*) and the three ICGs from *V. volvacea* (*SPRYp*, *Ras*, and *Vps26*).

From the functional point of view, the novel screened reference genes with the high conserved function perform basic cellular functions and participate in basic metabolic processes in cells, so they are suitable as novel reference genes to adapt to different experimental environments. Besides the conserved function, the expression abundance should also be considered when selecting the appropriate ICGs. We found that the traditional ICGs, such as *GAPDH* often have high abundance expression, which is suitable for the target gene with the high abundance expression. The use of very highly expressed genes as reference genes, such as *GAPDH* and 18S rRNA, will not accurately reflect the expression differences between lowly expressed genes [13]. When selecting the stable ICGs with the moderate or low abundance expression, the final calculation result of relative expression for the lowly expressed gene is more accurate. Therefore, in this study, on the premise of stable expression, the absolute expression values (the average RPKM) as well as the fluctuation range of each ICG were also detailed presented for reference in Table 1 and Table 2. It provides more scientific and useful information for the selection of appropriate ICGs.

We ranked the 12 ICGs tested in this study based on their evaluation value (stability index M values) of expression stability calculated by the 2 algorithms, geNorm and NormFinder. *RNB*, *V-ATP,* and *VAMP* were recommended to be the robust stable and reliable ICGs for RT-qPCR in filamentous fungi. The *Vps26*, *SPRYp*, *HUL4*, and *GAPDH* are ranked as the four least stable genes in *F. filiformis*, while *ACTB*, *SPRYp*, *Vps26*, *Cwf15*, and *DnaJ* are ranked as the five least stable genes in *N. crassa*. However, this does not mean that these genes cannot be used as ICGs, because their stability index M values (or stability value) are less than the 1.5 threshold, except for *Vps26* (its stability index M value or stability value > 1.5). The program geNorm sets the threshold of stability value for stable gene expression at 1.5, it means that it is acceptable as an eligible ICG when the M value is less than 1.5 [30]. Additionally, in the case that several genes meet this basic condition (M < 1.5), the smaller the M value, the more stable the expression of ICG. This also suggests that it is acceptable to use the traditional housekeeping genes (such as *ACTB*, *β-TUB*, *GAPDH*) and other candidates (such as *SPRYp*, *Ras*, *HUL4*, *Cwf15*, and *DnaJ*, except for *Vps26*) as the ICGs as long as their M values are less than 1.5 under the particular experimental conditions, although they are not the best choices. In addition, it should be mentioned that the reagents and instruments may affect the RT-qPCR, mainly on the Ct value of a particular gene, due to the systematic deviation. However, it has been reported in the literatures that gene relative expression results exhibited good reproducibility among different RT-qPCR instruments, different reagents [35,36]. Probably because the systematic deviation from different RT-qPCR instruments is equal for each ICG and target gene, and could be eliminated in the calculation. At the same time, we also tested the repeatability and stability of some Ct values of *ACTB* and *β-TUB* from two different RT-qPCR instruments, the similar mean Ct and standard deviation also indirectly indicates that the effect of RT-qPCR instruments on the stability and selection of ICG could be negligible. Therefore, when selecting ICGs for RT-qPCR in the future, on the one hand, we should select the robust and universal ICGs as far as possible according to the expression stability test and evaluation results; on the other hand, we can select multiple ICGs at the same time to obtain the most accurate gene expression results. At least two ICGs are required according to the MIQE standard recommended by Bustin et al. [28]. In addition, it is also worth considering that the ICGs should have the similar expression level (the same magnitude order of RPKM) with the target genes, especially for the genes expressed in low to medium abundance.

In conclusion, the three novel ICGs *RNB*, *VAMP*, and *V-ATP* showed more robust stability in multiple ascomycetes and basidiomycetes datasets than traditional housekeeping genes. The identification of the novel reliable ICGs in this study makes an important contribution to the selection of better ICGs for RT-qPCR normalization in filamentous fungi.

## Figures and Tables

**Table 1:** The maximum fold change (MFC) and the average expression levels (RPKM) of the traditional housekeeping genes and reference genes identified from *Volvariella volvacea* ^#^.

Sample Type	Species	GSE No.	Traditional Housekeeping Genes						Reference Genes from Vv ^#^					
			*ACTB*		*β-TUB*		*GAPDH*		*SPRYp*		*Ras*		*Vps26*	
			MFC	RPKM	MFC	RPKM	MFC	RPKM	MFC	RPKM	MFC	RPKM	MFC	RPKM
Different nutritional resources	*Neurospora crassa*	GSE60004	1.1	528.7	1.9	244.1	6.0	1367.8	2.1	57.2	1.5	53.8	1.2	33.9
		GSE35227	3.7	3537.8	3.6	4393.9	5.1	35,633.7	5.9	358.8	3.7	469.0	1.9	459.6
		GSE44673	1.3	907.7	1.6	603.9	2.4	3931.2	2.7	68.4	1.3	62.4	1.4	64.3
		GSE52316	1.1	1040.2	2.1	498.9	2.2	2725.7	1.4	85.5	1.8	39.1	1.2	43.8
		GSE60986	2.7	1082.7	3.7	614.5	3.4	2309.1	3.4	23.4	2.2	39.3	1.4	34.8
		GSE68517	2.0	972.5	2.2	474.4	4.1	4434.0	4.1	62.4	3.5	50.5	3.0	42.3
		GSE36719	2.3	9441.1	3.0	4126.0	1.7	47,828.7	3.2	485.6	1.2	239.6	2.0	285.9
		GSE42692	2.1	2886.0	2.6	4401.9	3.9	29,295.9	2.1	328.9	1.1	332.2	1.2	395.7
		GSE51091	1.7	1068.4	1.6	675.1	3.5	3795.7	1.2	36.6	1.1	72.5	1.2	40.0
	*Trichoderma reesei*	GSE53629	1.1	19,940.5	1.5	3357.9	2.8	43,090.2	2.0	3869.0	1.2	1293.1	1.3	1464.1
	*Aspergillus nidulans*	GSE44100	1.1	663.7	1.5	287.6	1.4	881.7	1.1	81.2	1.7	92.3	1.1	41.3
Different development stages	*Flammulina filiformis*	Wang et al., 2015	2.8	1803.0	2.6	2067.8	19.7	1616.2	2.0	98.4	3.3	228.7	2.0	39.9
	*Agaricus bisporus*	GSE39569	2.5	3298.3	4.5	821.0	5.5	81,562.8	10.9	72.5	3.7	55,015.0	2.2	12,820.5
	*Neurospora crassa*	GSE41484	2.4	1434.4	1.6	681.5	5.3	4659.3	3.2	115.6	1.8	284.8	2.5	92.8
	*Fusarium graminearum*	GSE61865	2.7	849.2	5.3	230.2	4.3	2048.4	3.6	72.0	4.3	56.5	1.7	66.3
Different stresses	*Neurospora crassa*	GSE53013	1.6	1354.9	1.6	781.9	2.0	4027.3	2.7	18.1	2.5	47.2	1.9	41.4
		GSE52153	1.3	1493.3	1.6	792.9	1.5	4637.7	3.3	26.8	1.8	58.4	2.2	57.8
		GSE53534	1.8	591.9	2.5	357.0	2.4	16,553.0	2.0	22.0	2.8	15.5	1.7	16.1
	*Magnaporthe oryzae*	GSE57146	1.5	659.2	1.4	253.6	1.5	7283.7	1.2	41.4	1.8	61.5	1.4	28.4
Different strains	*Flammulina filiformis*	Wang et al., 2016	1.6	2234.2	1.8	1594.7	2.5	7846.6	1.6	107.8	3.1	164.7	1.4	24.9
	*Volvariella volvacea*	GSE43019	1.9	1840.7	1.7	9.7	1.7	5063.7	1.0	66.3	1.5	8.0	1.4	77.1
	*Aspergillus nidulans*	GSE63672	1.4	1343.5	2.3	764.4	7.9	4399.4	1.5	149.4	1.5	159.3	1.5	71.5
The reference range of RPKM value * **(Average RPKM)**			500–3550 (1479.5)		200–4500 (1274.2)		800–50,000 (11,115.7)		18–150 (66.9)		8–500 (126.8)		16–100 (48.0)	

MFC: the ratio of the maximum and minimum values observed within the dataset [32]. The yellow background cells represent the maximum fold change > 3. ^#^: The three reference genes identified from *V. volvacea* [15]. *: The reference range of RPKM value and average RPKM of genes was determined after the extreme values were removed according to 99% confidence interval of the Grubbs criterion [33].

**Table 2:** The maximum fold change (MFC) and the average expression levels (RPKM) of the novel internal control genes.

Sample Type	Species	GSE No.	Novel Internal Control Genes											
			*DnaJ*		*Cwf15*		*HUL4*		*VAMP*		*RNB*		*V-ATP*	
			MFC	RPKM	MFC	RPKM	MFC	RPKM	MFC	RPKM	MFC	RPKM	MFC	RPKM
Different nutritional resources	*Neurospora crassa*	GSE60004	1.4	31.8	2.0	37.8	1.2	75.9	2.1	76.3	1.4	166.8	1.6	138.9
		GSE35227	2.0	250.2	2.0	216.4	1.8	592.0	2.2	581.9	1.7	1226.0	1.9	1861.9
		GSE44673	1.4	46.4	1.2	35.8	1.1	140.1	1.3	98.1	1.5	308.2	1.3	294.2
		GSE52316	1.2	36.1	1.3	33.1	1.4	95.0	1.4	86.7	1.4	275.2	1.3	292.7
		GSE60986	1.5	27.6	2.1	19.8	1.8	55.4	1.4	76.1	1.8	118.0	2.1	125.8
		GSE68517	1.5	32.0	1.5	28.1	1.7	79.6	2.3	85.1	3.0	157.6	1.7	245.8
		GSE36719	1.3	226.8	1.2	185.1	1.7	571.6	1.2	593.8	1.3	1184.6	1.4	1521.0
		GSE42692	1.1	220.7	1.2	206.4	1.5	545.7	1.1	522.1	1.6	1312.8	1.6	1685.7
		GSE51091	1.2	38.4	1.2	30.0	1.3	70.3	1.1	98.8	1.1	138.7	1.3	111.5
	*Trichoderma reesei*	GSE53629	1.2	1935.6	1.2	288.4	1.3	4480.3	1.2	2305.2	1.4	14,098.2	1.3	5809.2
	*Aspergillus nidulans*	GSE44100	1.6	66.9	1.5	50.5	1.1	98.0	1.5	135.7	1.1	211.7	1.1	88.1
Different development stages	*Flammulina filiformis*	Wang et al., 2015	1.7	24.5	1.8	49.8	2.0	54.2	1.8	274.8	1.5	182.5	1.5	198.0
	*Agaricus bisporus*	GSE39569	1.4	4334.8	3.0	11,422.0	1.1	6239.8	2.4	2175.3	1.7	5820.0	1.4	10,573.8
	*Neurospora crassa*	GSE41484	1.3	59.9	1.3	40.5	1.3	91.1	1.7	265.1	1.6	213.3	1.7	263.8
	*Fusarium graminearum*	GSE61865	1.5	87.6	2.4	73.2	2.3	168.6	1.8	160.6	2.9	69.5	1.5	103.4
Different stresses	*Neurospora crassa*	GSE53013	1.6	25.2	1.6	30.8	2.2	49.5	1.5	81.1	1.9	87.1	2.1	101.9
		GSE52153	1.6	32.1	1.9	33.4	1.5	64.6	1.5	93.7	2.2	129.7	1.6	144.8
		GSE53534	1.4	22.2	1.5	30.2	1.7	30.9	1.5	46.2	1.7	53.6	1.6	79.5
	*Magnaporthe oryzae*	GSE57146	1.1	30.9	2.2	28.2	1.8	56.3	1.2	119.5	1.2	56.5	2.8	104.5
Different strains	*Flammulina filiformis*	Wang et al., 2016	1.9	24.1	1.6	44.6	1.5	70.3	1.2	217.1	1.8	163.2	1.3	151.4
	*Volvariella volvacea*	GSE43019	2.0	48.2	1.4	0.4	1.3	2.6	1.8	2.8	2.4	8.6	2.5	54.9
	*Aspergillus nidulans*	GSE63672	1.3	109.2	1.3	67.1	1.9	293.5	1.5	165.8	2.0	467.9	1.4	79.8
The reference range of RPKM value * **(Average RPKM)**			20–260 (72.0)		15–300 (72.8)		30–600 (160.3)		45–600 (189.1)		50–1350 (326.6)		50–1900 (382.4)	

MFC: the ratio of the maximum and minimum values observed within the dataset [32]. *: The reference range of RPKM value and average RPKM of genes was determined after the extreme values were removed according to 99% confidence interval of the Grubbs criterion [33].

**Table 3:** The predicted function of the 12 reference ICGs.

Gene Symbol	Gene Name	Pfam Annotation	Function
*ACTB*	*β*-actin	Actin (PF00022)	Polymerize into microfilament and constitute major component of the cytoskeleton
*β-TUB*	*β*-tubulin	Tubulin/FtsZ family, GTPase domain (PF00091)	Be involved in cell division and constitutes the main component of microtubules
*GAPDH*	Glyceraldehyde 3-phosphate dehydrogenase	Glyceraldehyde 3-phosphate dehydrogenase (PF02800)	Be involved in glycolysis and gluconeogenesis
*SPRYp*	SPRY-domain-containing protein	SPRY domain (PF00622)	Be involved in RNA processing and histone H3 methylation regulatory signaling pathways and regulates nutrient transport
*Ras*	Ras-2 protein	Ras family (PF00071)	Regulate cytoskeletal integrity, proliferation, apoptosis and cell migration
*Vps26*	Vacuolar protein sorting protein 26	Vacuolar protein sorting-associated protein 26 (PF03643)	Be involved in protein trafficking and regulate vesicular protein sorting
*Cwf15*	Pre-mRNA-splicing factor cwc15	Cwf15/Cwc15 cell cycle control protein (PF04889)	Be involved in pre-mRNA splicing
*DnaJ*	ER associated DnaJ chaperone	DnaJ domain (PF00226)	Be involved in folding of nascent proteins and regulate the responses to stress
*HUL4*	E3 ubiquitin-protein ligase NEDD4	HECT-domain (ubiquitin-transferase) (PF00632)	Constitute ubiquitin-protein ligases and participate in protein ubiquitination hydrolysis
*VAMP*	ATP-binding cassette, subfamily B (MDR/TAP), member 1	MSP (Major sperm protein) domain (PF00635)	Constitute cell cytoskeleton, with related vesicle-associated membrane protein and participate in vesicle fusion
*RNB*	Exosome complex exonuclease DIS3/RRP44	RNB domain (PF00773)	Constitute ribonuclease II and involved in the mRNA degradation pathway
*V-ATP*	V-type H^+^-transporting ATPase subunit A	ATP synthase alpha/beta family, nucleo-tide-binding domain (PF00006)	Driven proton pump and involved in protein transport, active transport of metabolites and homeostasis

**Table 4:** The stability analysis of 12 ICGs candidates using NormFinder in *F. filiformis* and *N. crassa*.

Sample Sets	*F.filiformis* Samples Set (A)		*N. crassa* Samples Set (B)		Comprehensive Analysis A and B	
Rank	Gene Name	Stability Value	Gene Name	Stability Value	Gene Name	Stability Value
1	*RNB*	0.110	*RNB*	0.387	*V-ATP*	0.175
2	*V-ATP*	0.114	*VAMP*	0.398	*RNB*	0.261
3	*VAMP*	0.353	*V-ATP*	0.454	*β-TUB*	0.266
4	*DnaJ*	0.427	*HUL4*	0.501	*VAMP*	0.348
5	*β-TUB*	0.440	*β-TUB*	0.512	*Ras*	0.546
6	*Cwf15*	0.536	*Ras*	0.542	*GAPDH*	0.681
7	*ACTB*	0.745	*GAPDH*	0.563	*HUL4*	0.699
8	*Ras*	0.909	*DnaJ*	0.639	*DnaJ*	1.098
9	*GAPDH*	1.104	*Cwf15*	0.828	*Cwf15*	1.350
10	*HUL4*	1.185	*Vps26*	0.835	*SPRYp*	1.426
11	*SPRYp*	1.251	*SPRYp*	0.948	*ACTB*	1.501
12	*Vps26*	1.610	*ACTB*	1.365	*Vps26*	1.507

## Data Availability

All experimental data in this study will be made available upon reasonable request from readers.

## Supplementary Materials

The following supporting information can be downloaded at: https://www.mdpi.com/article/10.3390/jof8090952/s1, Table S1: The 10 samples of *Flammulina filiformis* and 8 samples of *Neurospora crassa* involved in this study; Table S2: The corresponding accession number of 12 ICGs in *F. filiformis* and *N. crassa*; Table S3: Descriptions of 12 ICGs candidates and the primers used in *F. filiformis*; Table S4: Descriptions of 12 ICGs candidates and the primers used in *N. crassa*; Table S5. Raw Ct values of *ACTB* and *β-TUB* genes of *N. crassa* in WT strains on LC96 and CFX96 instruments; Figure S1. Raw Ct values bar graph of *ACTB* and *β-TUB* genes of *N. crassa* in WT strains on LC96 and CFX96 instruments, respectively.
