# Supplementary material for: Genome-Wide Screening and Stability Verification of the Robust Internal Control Genes for RT-qPCR in Filamentous Fungi
Source: J Fungi (Basel). 2022 Sep 10;8(9):952. doi: 10.3390/jof8090952 (PMC9504127; doi:10.3390/jof8090952)
Supplement: Supplementary file 1 [file jof-08-00952-s001.zip › jof-1891045-supplementary Revised.pdf]

Supplementary materials:
Genome-Wide Screening and Stability Verification of the Robust Internal Control Genes for RT-qPCR in Filamentous Fungi

Yayong Yang[1,2,†], Xinyu Xu[1,2,†], Zhuohan Jing[1,2], Jun Ye[1,2], Hui Li[3], Xiaoyu Li[1,2], Lei Shi[1,2], Mengyu Chen[1,2], Tengyun Wang[1,2], Baogui Xie[2], Yongxin Tao[1,2,*]

[1] College of Horticulture, Fujian Agriculture and Forestry University, Fuzhou 350002, China
[2] Mycological Research Center, College of Life Sciences, Fujian Agriculture and Forestry University, Fuzhou 350002, China
[3] Institute of Cash Crops, Hebei Academy of Agriculture and Forestry Sciences, Shijiazhuang 050051, China
* Correspondence: taoyongxinmuse@163.com; Tel.: +86-0591-83789281
† These authors contributed equally to this work.

Table S1. The 10 samples of *Flammulina filiformis* and 8 samples of *Neurospora crassa* involved in this study

NO.	Materials	Abbreviation	Notes
1	Mycelia	MY	Heterokaryon
2	Aerial hyphal knot	AHK	Heterokaryon
3	Primordia	PR	Fruiting body of strain
4	Bud	BUD	Fruiting body of strain
5	Pileus in young fruiting body	YFP	Fruiting body of strain
6	Stipe in young fruiting body	YFS	Fruiting body of strain
7	Pileus in elongation stage	ELP	Fruiting body of strain
8	Stipe in elongation stage	ELS	Fruiting body of strain
9	Pileus in maturation stage	MAP	Fruiting body of strain
10	Stipe in maturation stage	MAS	Fruiting body of strain
**NO.**	**Abbreviation**	**Treatment condition**	
11	WT	The temperature of 25°C	
12	ΔSpds	The temperature of 25°C	
13	BT	The temperature of 25°C, the blue light treatment for 3 d, and the light intensity of 3 μmol·m^{-2}·s^{-1}	
14	BS	The temperature of 25°C, the blue light stress for 3 h, and the light intensity of 3 μmol·m^{-2}·s^{-1}	
15	CS	Cold stress 4 °C, cultured for 30 min	
16	HS	Heat stress 42 °C, cultured for 30 min	
17	JA	The concentration of 0.01 mmol/L	
18	H_2O_2	The concentration of 5 mmol/L	
The two different combinations of sample sets:			
A	*F. filiformis* samples	Samples of 1, 2, 3, 4, 5, 6, 7, 8, 9 and 10	
B	*N. crassa* samples	Samples of 11, 12, 13, 14, 15, 16, 17 and 18	

Table S2. The corresponding accession number of 12 ICGs *F. filiformis* and *N. crassa*

Gene symbol	Gene name	Accession number in Ff-L11	Accession number in Nc-OR74A
ACTB	*β*-actin	OP354401	NCU04173
beta-TUB	*β*-tubulin	OP354402	NCU04054
GAPDH	Glyceraldehyde 3-phosphate dehydrogenase	OP354403	NCU01528
SPRYp	SPRY-domain-containing protein	OP354404	NCU03678
Ras	Ras-2 protein	OP354405	NCU03616
Vps26	Vacuolar protein sorting protein 26	OP354406	NCU02743
Cwf15	Pre-mRNA-splicing factor cwc15	OP354407	NCU00335
DnaJ	ER associated DnaJ chaperone	OP354408	NCU03335
HUL4	E3 ubiquitin-protein ligase NEDD4	OP354409	NCU03947
VAMP	ATP-binding cassette, subfamily B (MDR/TAP), member 1	OP354410	NCU08956
RNB	Exosome complex exonucleaseDIS3/RRP44	OP354411	NCU01197
V-ATP	V-type H^+-transporting ATPase subunit A	OP354412	NCU01207

Table S3. Descriptions of 12 ICGs candidates and the primers used in *F. filiformis*

Gene symbol	Gene name	Primer	Sequences (5′–3)	Product size (bp)	Annealing temperature (°C)
ACTB	*β*-actin	F5510QF<br>F5510QR	GATCGTATGCAGAAGGAGTTGACAC<br>CCACTCTCGTCGTACTCTTGCTTG	211	62
β-TUB	*β*-tubulin	F346QF<br>F346QR	CAAATGCAGAACGTCCAGAAC<br>GTGAACTCCATCTCGTCCATAC	242	62
GAPDH	Glyceraldehyde 3-phosphate dehydrogenase	F5082QF<br>F5082QR	GTTTCCGTTGTTGACCTTGTGTGCC<br>CGAAGTTGCCGTTGAGCTGGATAC	118	62
SPRYp	SPRY-domain-containing protein	F5226QF<br>F5226QR	CACAGTAATATCTCCAGACGCGATC<br>TCCTACCACTCCTTCGCCTGTTG	237	62
Ras	Ras-2 protein	F5609QF<br>F5609QR	TCAATGCGACGAGTAAAGAGAGG<br>CATAGGTCCCACATCTACATTTCG	233	62
Vps26	Vacuolar protein sorting protein 26	F898QF<br>F898QR	CGAGGCGAAACTATCCCAATAC<br>TATCGTCTGTTCTCCTCGTCTAC	134	62.5
Cwf15	Pre-mRNA-splicing factor cwc15	F7920QF<br>F7920QR	CTGAAAGAGCTGGAGAAGATCAA<br>CAGCGTCTCTTTACGGAGAAA	206	62
DnaJ	ER associated DnaJ chaperone	F2761QF<br>F2761QR	CTCATCCTCTTTGCCCTTTCA<br>CTGTCGGGTTGACGAAGTATT	157	62
HUL4	E3 ubiquitin-protein ligase NEDD4	F6860QF<br>F6860QR	CCGAGGAGAACAAGAAGGAATAC<br>ACTTGGTCCAATCGTCCATATC	198	62
VAMP	ATP-binding cassette, subfamily B (MDR/TAP), member 1	F7311QF<br>F7311QR	GAGAAGATCAAAGAGGCTGAGG<br>GAAGAGGTAGGTCGTGATGAATAC	246	62
RNB	Exosome complex exonuclease DIS3/RRP44	F2240QF<br>F2240QR	TTGTTCCTCTGTGTTCTCATCTC<br>GACATCCTTCGTGGACCAATAG	216	62
V-ATP	V-type H^+-transporting ATPase subunit A	F5791QF<br>F5791QR	GCAGAATGGTATGAGCGAGTAT<br>TCTGGGACAGCTTGAACATAAG	173	62

Table S4. Descriptions of 12 ICGs candidates and the primers used in *N. crassa*

Gene symbol	Gene name	Primer	Sequences (5′–3)	Product size (bp)	Annealing temperature (°C)
ACTB	*β*-actin	NCU04173QF<br>NCU04173QR	CTCCATCATGAAGTGCGATGT<br>TTCATGGAAGAAGGAGCAAGAG	138	56
β-TUB	*β*-tubulin	NCU04054QF<br>NCU04054QR	GTCTCCATGAAGGAGGTTGAG<br>GAAGGTGGAGGACATCTTGAG	141	58
GAPDH	Glyceraldehyde 3-phosphate dehydrogenase	NCU01528QF<br>NCU01528QR	GGTTGTCTCTTCCGACATGAA<br>GCATCGACCTTGGAGATGTAG	168	57
SPRYp	SPRY-domain-containing protein	NCU03678QF<br>NCU03678QR	TTCCGAGCAAGGCAGTATTC<br>TGATCGTGTGCGGTTATGAG	151	55
Ras	Ras-2 protein	NCU03616QF<br>NCU03616QR	CCTGTGCCGATTATGCTAGTG<br>TCAACATTTGTGCGTGTCTTTG	140	56
Vps26	Vacuolar protein sorting protein 26	NCU02743QF<br>NCU02743QR	CCGCATCTACTTCTTGCTTGTA<br>GGAATGGTCTCTCCTCTTGATG	162	57
Cwf15	Pre-mRNA-splicing factor cwc15	NCU00335QF<br>NCU00335QR	AGACTCCTCCGATTCTGATTCC<br>CTTCTCGCGCTGTTCTTCTTC	157	58
DnaJ	ER associated DnaJ chaperone	NCU03335QF<br>NCU03335QR	CAAACGACGTCAGGCATTATTC<br>GGCCAACTTCTCTTGATCTGTA	170	56
HUL4	E3 ubiquitin-protein ligase NEDD4	NCU03947QF<br>NCU03947QR	GGTAGTGATGGTCCTCGTAGA<br>CATAGTCTCCTCCACAGCAATC	156	58
VAMP	ATP-binding cassette, subfamily B (MDR/TAP), member 1	NCU08956QF<br>NCU08956QR	CCATCGCATCTCTCCAGAAC<br>GAAGATATAGGCCAGCAGGAAG	203	58
RNB	Exosome complex exonuclease DIS3/RRP44	NCU01197QF<br>NCU01197QR	CTCTGACATCAGAAAGGGTATGG<br>GGGTACGTGAAATGAGTGTAGAG	141	58
V-ATP	V-type H^+-transporting ATPase subunit A	NCU01207QF<br>NCU01207QR	GATTTCTCTGATCCCGTCACTTC<br>GGTACTCCCTCTCATACCACTT	163	58

Table S5. Raw Ct values of *ACTB* and *β-TUB* genes of *N. crassa* in WT strains on LC96 and CFX96 instruments

**Gene**	***ACTB***		***β-TUB***	
**Instrument**	LC96	CFX96	LC96	CFX96
**Raw Ct value**	27.25	27.18	22.51	24.55
	27.13	27.93	22.13	23.77
	27.06	27.12	21.94	24.05
	28.59	28.60	23.86	22.99
	27.94	28.69	23.66	23.32
	27.87	28.59	23.18	22.79
	28.01	28.98	21.98	22.42
	28.16	29.05	21.76	23.15
	28.22	29.04	22.44	22.68
**Mean Ct**	27.80333333	28.35333333	22.60666667	23.30222222
**Standard deviation**	0.505261209	0.719320667	0.732378166	0.656908209
***P* value**	0.0958		0.0628	

Notice: LC96: LightCycler® 96 Instrument; CFX96: Bio–Rad CFX96 Real-Time PCR Detection System. *P* values were derived from unpaired t tests.

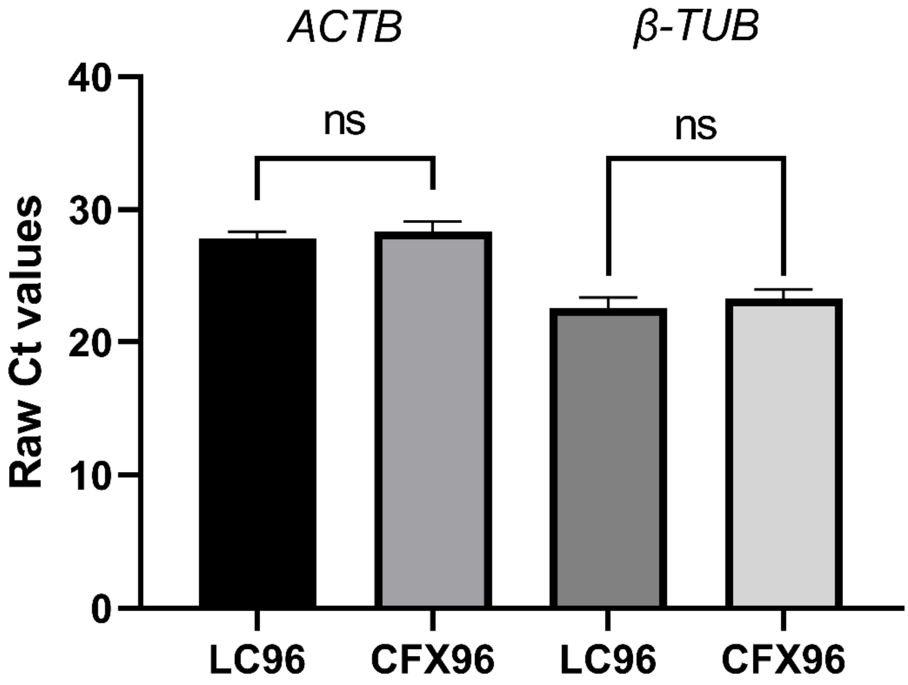

Figure S1. Raw Ct values bar graph of *ACTB* and *β-TUB* genes of *N. crassa* in WT strains on LC96 and CFX96 instruments, respectively. Average Ct value of three biological replicates and three technical replicates were shown on the Y-axis. The two different instruments LC96 (LightCycler® 96 Instrument) and CFX96 (Bio–Rad CFX96 Real-Time PCR Detection System) were shown on the X-axis. The values are the means ± SD of three independent experiments. (Student's t test: ns: no significant).
